# Alleviative effects from boswellic acid on acetaminophen-induced hepatic injury

**DOI:** 10.7603/s40681-016-0009-1

**Published:** 2016-05-09

**Authors:** Lung-Che Chen, Li-Hong Hu, Mei-Chin Yin

**Affiliations:** 1 Department of Otolaryngology, Taipei Medical University Hospital, 110 Taipei, Taiwan; 2 Shanghai Research Center for the Modernization of Traditional Chinese Medicine, Shanghai Institute of Materia Medica, Chinese Academy of Sciences, 201203 Shanghai, China; 3 Department of Nutrition, China Medical University, 404 No. 91, Hsueh-Shih Road, Taichung, China

**Keywords:** Acetaminophen, Boswellic acid, CYP2E1, JNK, TLRs, NF-κB

## Abstract

Protective effects of boswellic acid (BA) against acetaminophen (APAP)-induced hepatotoxicity in Balb/ cA mice were examined. BA, at 0.05 or 0.1%, was supplied for 4 weeks. Acute liver injury was induced by APAP treatment. Results showed that BA intake increased hepatic BA bioavailability. APAP treatment decreased glutathione (GSH) level, increased reactive oxygen species (ROS) and oxidized glutathione (GSSG) production; and lowered activity and protein expression of glutathione reductase (GR) and heme oxygenase (HO)-1 in liver. BA intake at both doses alleviated subsequent APAP-induced oxidative stress by retaining GSH content, decreasing ROS and GSSG formations, reserving activity and expression of GR and HO-1 in liver, and lowering hepatic cytochrome P450 2E1 activity and expression. APAP treatment enhanced hepatic levels of interleukin-6, tumor necrosis factor-alpha and monocyte chemoattractant protein-1. BA pre-intake diminished APAP-induced release of those inflammatory cytokines and chemokines. APAP upregulated hepatic protein expression of toll-like receptor (TLR)-3, TLR-4, MyD88, nuclear factor kappa B (NF-κB) p50, NF-κB p65 and JNK. BA pre-intake at both doses suppressed the expression of NF-κB p65 and p-JNK, and only at 0.1% down-regulated hepatic TLR-3, TLR-4 and MyD88 expression. APAP led to obvious foci of inflammatory cell infiltration in liver, determined by H&E stain. BA intake at both doses attenuated hepatic inflammatory infiltration. These findings support that boswellic acid is a potent hepatoprotective agent.

## 1. Introduction

Acetaminophen (APAP, also called paracetamol) is a widely used analgesic and antipyretic drug. It is metabolized by hepatic cytochrome P450 system, especially CYP2E1, which leads to the overproduction of reactive free radicals and n-acetyl-p-benzoquinoneimine (NAPQI) [1, 2]. It is well known that high doses of APAP deplete hepatic glutathione (GSH) because NAPQI reacts rapidly with GSH, which consequently diminishes antioxidant defense, enhances oxidation stress, impairs liver functions, causes hepatocyte necrosis, and even promotes liver failure or death [3, 4]. In addition, the increased release of monocyte chemoattractant protein (MCP)-1, interleukin (IL)-6 and tumor necrosis factor (TNF)-alpha, due to the stimulation of oxidative stress from APAP, also contributes to the progression of APAP evoked hepatotoxicity [5, 6]. Thus, any agent with the ability to reserve GSH, inhibit CYP2E1 activity and/or lower inflammatory cytokines and chemokines may potentially attenuate APAP related liver injury.

Toll-like receptors (TLRs) are pattern recognition receptors mainly responsible for immuno modulation. The activation of TLR-3 and TLR-4 has been implicated in APAP-induced hepatic damage because both TLRs could stimulate the production of adaptor proteins including MYD88 and nuclear factor kappa B (NF-κB), which in turn promote the generation of oxidants and inflammatory cytokines and chemokines [7, 8]. Furthermore, Cavassani *et al*. [9] reported that TLR-3 activation enhanced JNK phosphorylation in APAP injured livers. Salama *et al*. [8] indicated that TLR-4 blocker was a potential therapeutic agent to improve APAP caused organ failure in mice. Thus, the inhibition upon TLR-3, TLR-4, NF-κB and/or JNK may be a strategy for alleviating APAP-induced hepatotoxicity.

**Fig. 1:**
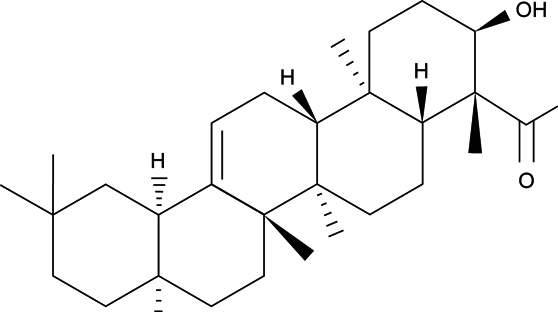
Structure of boswellic acid.

Boswellic acid (BA, Figure 1), a pentacyclic triterpene, naturally occurs in many *Boswellia* species including *Boswellia carteri, Boswellia serrata* and *Boswellia sacra* [10]. It is reported that BA could benefit the amelioration of chronic inflammatory diseases like arthritis, cerebral edema and chronic bowel diseases *via* its anti-oxidative and anti-inflammatory activities [11]. The study of Gayathri *et al*. [12] revealed that BA exhibited anti inflammatory activities in human peripheral blood mononuclear cells (PBMCs) through lowering the release of inflammatory cytokines. So far, the impact of BA upon APAP induced hepatic inflammatory reactions and signaling pathways remains known. Our previous study revealed that dietary intake of BA increased its bioavailability in liver of mice [13]. Thus, it is highly possible that BA intake or supplement could increase its accumulation in liver, and further enhance hepatic defensive capability to prevent or attenuate liver injury.

The purpose of this animal study was to examine the protective effects and action modes of BA at various doses upon liver of acetaminophen treated mice. The influence of this compound upon GSH, ROS and cytokines variation, CYP2E1 activity and protein expression of associated factors such as NF-κB, TLR-3 and TLR-4 was evaluated. Furthermore, histological analysis was processed in order to provide more solid evidence to support the benefit of using BA as a hepatic protective agent.

## 2. Materials and methods

### 2.1. Materials

Boswellic acid (BA, 98.5%) was synthesized and provided by Shanghai Institute of Materia Medica, Chinese Academy of Sciences, China. APAP (98%) was purchased from Sigma Chemical Co. (St. Louis, MO, USA).

### 2.2. Animals and diets

Five- to six-week-old male Balb/cA mice were obtained from National Laboratory Animal Center (National Science Council, Taipei City, Taiwan). Mice were housed on a 12-h light-12-h dark schedule, and fed with water and mouse standard diet (PMI Nutrition International LLC, Brentwood, MO, USA). Use of the mice was reviewed and approved by the China Medical University animal care committee (104-305).

### 2.3. Experimental design

BA at 0.05 or 0.1 g was mixed with 99.95 or 99.9 g powder diet to prepare 0.05 and 0.1% BA diets. Mice were divided into five groups: normal group (normal diet); BA group (0.1% BA diet), APAP group (normal diet plus APAP treatment); BA-low- APAP group (0.05% BA diet plus APAP treatment); BA-high- APAP group (0.1% BA diet plus APAP treatment). After 4 weeks supplement, normal and BA groups were sacrificed, and the other three groups were treated by a single intraperitoneal injection of APAP (400 mg/kg body weight). APAP was dissolved in phosphate-buffered saline (PBS). Mice were killed with carbon dioxide after 24 h. The mortality of mice due to APAP injection was zero. After sacrificed, liver from each mouse was collected and weighted. There was no mice die before sacrificed. Blood was also collected, and serum was separated from erythrocyte immediately. Liver tissue, 0.2 g, was homogenized on ice in 2 ml phosphate buffer (pH 7.2), and the filtrate was collected. The protein concentration of serum and liver filtrate was determined by a commercial assay kit (Pierce Biotechnology Inc., Rockford, IL, USA) with bovine serum albumin as standard.

### 2.4. Determination of BA content in liver

The HPLC method of Lozano-Mena *et al*. [14] was used to analyze the content of BA in liver. Liver homogenate, 100 μl, was treated with ethyl acetate for extraction. The organic layers from 3-time extractions were combined and dried by nitrogen; the residue was reconstituted in 200 μl methanol/water (75:25, v/v) and centrifuged at 12,000 × g for 5 min at 4°C. Agilent 1100 series HPLC system (Agilent Corp, Waldbronn, Germany) equipped with a C18 reversed-phase column (100 mm × 4 mm, 3 μm, Thermo Electron, Bellafonte, PA, USA) was used. BA quantification was performed using the external standard method. The limit of detection was 0.1 mg/g tissue. The relative standard deviations of precision and accuracy were less than 5.2%.

### 2.5. Alanine aminotransferase (ALT), aspartate aminotransferase (AST) and C-reactive protein (CRP) analyses

Serum activities of ALT and AST were determined by using commercial assay kits (Randox Laboratories Ltd., Crumlin, UK). CRP level (mg/l) was measured by an ELISA kit (Anogen, ON, Canada).

### 2.6. Assays for GSH, oxidized glutathione (GSSG) and reactive oxygen species (ROS) levels, and activity of glutathione peroxidase (GPX) and glutathione reductase (GR)

Liver tissue was homogenized with cold PBS containing 0.05% Tween 20 and 1 mM EDTA. After centrifuging, supernatants were used for measurements. GSH and GSSG concentrations (nmol/mg protein) in liver were determined by commercial colorimetric GSH and GSSG assay kits (OxisResearch, Portland, OR, USA). ROS level was quantified by using 2’, 7’-dichlorofluorescein diacetate. Fluorescence value was measured by using a fluorescence microplate reader at excitation and emission wavelengths of 485 and 530 nm, respectively. Relative fluorescence unit (RFU) was the difference in fluorescence values obtained at time 0 and 5 min. Results are expressed as RFU/mg protein. The activity (U/mg protein) of GPX and GR was assayed by using commercial kits purchased from EMD Biosciences Co. (San Diego, CA, USA).

### 2.7. Cytokines measurements

Hepatic levels of IL-6, TNF-alpha and MCP-1 were measured by using cytoscreen immunoassay kits (BioSource International, Camarillo, CA, USA). The sensitivity of assay with the detection limit was 5 pg/ml for IL-6, and 10 pg/ml for TNF-alpha and MCP-1.

**Tabel 1.** Body weight (BW, g/mouse), water intake (WI, ml/mouse/day), feed intake (FI, g/mouse/day), liver weight (LW, mg/mouse) and hepatic BA content (mg/g) in mice with BA intake at 0, 0.05 or 0.1% for 4 weeks, and followed by APAP treatment. Normal groups had neither BA nor APAP treatment. BA groups had 0.1% BA intake and without APAP treatment. Values are mean ± SD, n = 10.

	normal	BA, 0.1	APAP	BA, 0.05 + APAP	BA, 0.1 + APAP
BW	26.2 ± 1.7^a^	26.3 ± 1.5^a^	25.9 ± 2.0^a^	26.0 ± 1.3^a^	26.5 ± 1.6^a^
WI	1.9 ± 0.4^a^	2.0 ± 0.3^a^	1.8 ± 0.6^a^	2.1 ± 0.5^a^	2.3 ± 0.7^a^
FI	2.2 ± 0.6^a^	2.1 ± 0.4^a^	2.3 ± 0.5^a^	2.0 ± 0.3^a^	2.2 ± 0.4^a^
LW	1.40 ± 0.09^a^	1.35 ± 0.1^a^	1.29 ± 0.11^a^	1.33 ± 0.05^a^	1.37 ± 0.12^a^
BA	-*,^a^	0.45 ± 0.1^a^	-^a^	-^a^	0.28 ± 0.08^a^

*Means too low to be detected.

^a,b^ Means in a row without a common letter differ, *P* < 0.05.

**Fig. 2:**
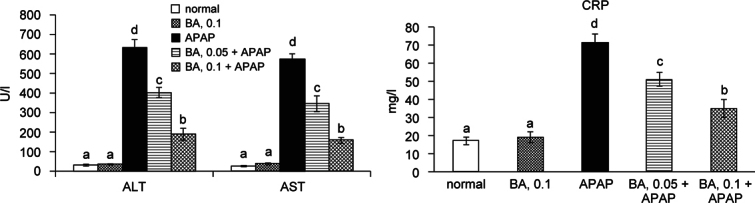
Serum ALT, AST and CRP levels in mice with BA intake at 0, 0.05 or 0.1% for 4 weeks, and followed by APAP treatment. Normal groups had neither BA nor APAP treatment. BA groups had 0.1% BA intake and without APAP treatment. A representative image is shown for each group. Values are mean ± SD, n = 10. ^a-d^Means among bars without a common letter differ, *P* < 0.05.

### 2.8. Measurement of CYP2E1 activity

The activity of CYP2E1 in freshly prepared liver microsome was estimated by colorimetrically measuring the formation of 4-nitrocatechol, a product from p-nitrophenol hydroxylation catalyzed specifically by CYP2E1. The protein concentration of CYP2E1 was measured by ELISA, and a rabbit anti-CYP2E1 antibody (Calbiochem, Inc., San Diego, CA, USA) was used for detection. The formed 4-nitrocatechol was expressed as nmol/mg protein.

### 2.9. Western blot analyses

Hepatic tissue, 40 mg, was homogenized in buffer containing protease-inhibitor cocktail purchased from Sigma-Aldrich Chemical Co. (St. Louis, MO, USA) and 0.5% Triton X-100. Homogenate was then mixed with buffer (60 mmol/L Tris-HCl, 2% β-mercaptoethanol and 2% SDS, pH 7.2), and followed by boiling for 5 min. Protein sample at 40 μg was electrophoresed on 10% SDS-polyacrylamide gel, and further transferred onto nitrocellulose membranes (Millipore, Bedford, MA, USA) for 1 h. After blocking with a protein solution containing 5% skim milk for 1 h, membranes were treated with monoclonal antibody (Boehringer- Mannheim, Indianapolis, IN, USA) against heme oxygenase (HO)-1, CYP2E1 (1:1000), NF-κB p50, NF-κB p65, JNK (1:500), TLR-3, TLR-4, MyD88 (1:2000) at 4ºC overnight, and followed by incubating with horseradish peroxidase-conjugated antibody at room temperature for 3.5 h. Glyceraldehyde-3-phosphate dehydrogenase (GAPDH) was used as a loading control, and the bands were quantified by an ATTO image analyzer (Tokyo, Japan).

### 2.10. Histological Analyses

Partial liver tissue from each mouse was fixed in 10% phosphatebuffered formalin, and embedded in paraffin. Paraffin section at 5 mm thickness was cut and processed with hematoxylin-eosin (H&E) stain, and followed by examining under a light microscope for histological analysis. The severity of hepatic inflammatory injury was assayed according to the Ishak scoring system [15]. The injured area was quantified by two independent investigators in a double-blind manner.

### 2.11. Statistical analyses

The effect of each treatment was analyzed from 10 mice (n = 10) in each group. Data were reported as means ± standard deviation (SD), and subjected to analysis of variance. Differences among means were determined by the Least Significance Difference Test with significance defined at P < 0.05.

## 3. Results

### 3.1. BA decreased serum ALT, AST and CRP levels in APAP treated mice

BA intake at 0.1% increased hepatic BA content, and did not affect body weight, feed intake, water intake and liver weight (*P* > 0.05, Table 1). Serum levels of ALT, AST and CRP are presented in Figure 2. Without APAP treatment, 0.1% BA intake did not alter any measurements (*P* > 0.05). The intake of BA at both doses alleviated subsequent APAP-induced elevation of ALT, AST and CRP levels in serum (*P* & 0.05).

**Tabel 2.** Hepatic level of GSH (nmol/mg protein), GSSG (nmol/mg protein), ROS (RFU/mg protein); and hepatic activity (U/mg protein) of GPX and GR in mice with BA intake at 0, 0.05 or 0.1% for 4 weeks, and followed by APAP treatment. Normal groups had neither BA nor APAP treatments. BA groups had 0.1% BA intake and without APAP treatment. Values are mean ± SD, n = 10.

	normal	BA, 0.1	APAP	BA, 0.05 + APAP	BA, 0.1+ APAP
GSH	23.2 ± 1.5^d^	24.0 ± 1.1^d^	12.5 ± 1.8^a^	15.1 ± 1.0^b^	18.5 ± 1.3^c^
GSSG	0.23 ± 0.07^a^	0.21 ± 0.04^a^	1.18 ± 0.12^d^	0.90 ± 0.09^c^	0.65 ± 0.11^b^
ROS	0.19 ± 0.05^a^	0.16 ± 0.04^a^	1.39 ± 0.15^d^	1.02 ± 0.08^c^	0.59 ± 0.14^b^
GPX	32.6 ± 1.8^a^	32.1 ± 1.4^a^	36.4 ± 2.7^b^	34.8 ± 2.0^b^	35.0 ± 1.3^b^
GR	31.0 ± 1.5^d^	32.9 ± 1.2^d^	19.5 ± 1.0^a^	23.1 ± 0.8^b^	27.4 ± 1.3^c^

*a-d*Means in a row without a common letter differ, *P* < 0.05.

**Fig. 3:**
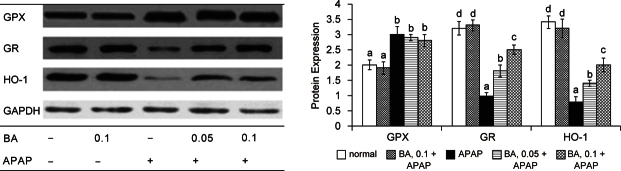
Hepatic GPX, GR and HO-1 expression in mice with BA intake at 0, 0.05 or 0.1% for 4 weeks, and followed by APAP treatment. Normal groups had neither BA nor APAP treatments. BA groups had 0.1% BA intake without APAP treatment. A representative image is shown for each group. Values are mean ± SD, n = 10. ^a-d^Means among bars without a common letter differ, *P* < 0.05.

### 3.2. BA attenuated oxidative and inflammatory stress in APAP treated mice

**Fig. 4:**
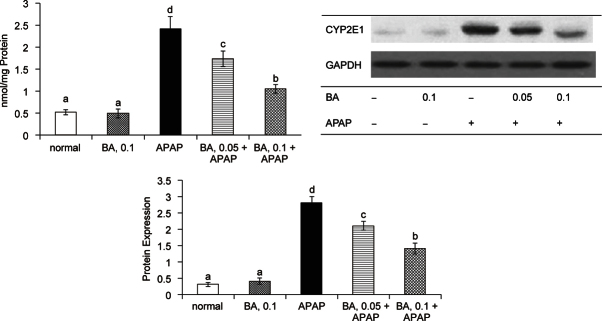
Liver microsomal CYP2E1 activity, determined as 4-nitrocatechol (nmol/mg protein), and protein expression in mice with BA intake at 0, 0.05 or 0.1% for 4 weeks, and followed by APAP treatment. Normal groups had neither BA nor APAP treatment. BA groups had 0.1% BA intake without APAP treatment. A representative image is shown for each group. Values are mean ± SD, n = 10. ^a-d^ Means among bars without a common letter differ, *P* < 0.05.

As shown in Table 2, APAP treatment significantly decreased GSH content, and raised GSSG and ROS levels in liver (*P* & 0.05); but BA pre-intake attenuated APAP-induced GSH depletion and decreased hepatic GSSG and ROS production (*P* & 0.05). APAP raised GPX activity and reduced GR activity in liver (*P* & 0.05). BA intake failed to change hepatic GPX activity (*P* > 0.05); but significantly maintained hepatic GR activity (*P* & 0.05). As shown in Figure 3, APAP treatment significantly increased GPX protein expression; and reduced protein expression of GR and HO-1 (*P* & 0.05). BA intake did not affect GPX protein expression (*P* & 0.05); but significantly retained protein expression of GR and HO-1 in liver (*P* & 0.05). APAP treatment enhanced CYP2E1 activity and expression (Figure 4, *P* & 0.05); however, BA intake significantly suppressed subsequent APAP-induced elevation of CYP2E1 activity and protein expression (*P* & 0.05). APAP treatment also significantly increased hepatic release of TNF-alpha, IL-6 and MCP-1 (Table 3, *P* & 0.05). BA intake lowered APAP-induced hepatic production of these cytokines (*P* & 0.05).

### 3.3. BA regulated signaling pathways in APAP treated mice

**Fig. 5:**
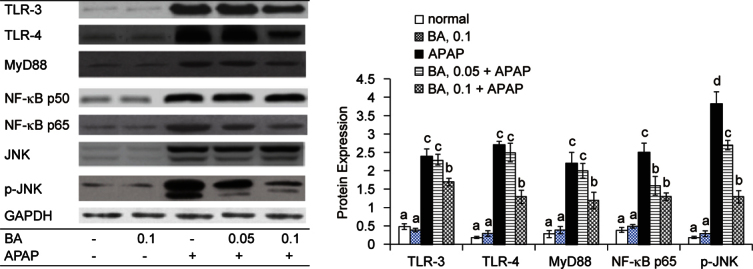
Hepatic protein expression of TLR-3, TLR-4, MyD88, NF-κB p50, NF-κB p65, JNK and p-JNK in mice with BA intake at 0, 0.05 or 0.1% for 4 weeks, and followed by APAP treatment. Normal groups had neither BA nor APAP treatment. BA groups had 0.1% BA intake without APAP treatment. A representative image is shown for each group. Values are mean ± SD, n = 10. ^a-d^ Means among bars without a common letter differ, *P* < 0.05.

APAP up-regulated hepatic protein expression of TLR-3, TLR-4, MyD88, NF-κB p50, NF-κB p65, JNK and p-JNK (Figure 5, *P* & 0.05). BA pre-intake at both doses down-regulated the expression of NF-κB p65 and p-JNK (*P* & 0.05), and only at high dose suppressed hepatic TLR-3, TLR-4 and MyD88 expression (*P* & 0.05). BA did not alter NF-κB p50 expression (*P* > 0.05).

### 3.4. BA improved inflammatory cell infiltration in APAP treated mice

**Fig. 6:**
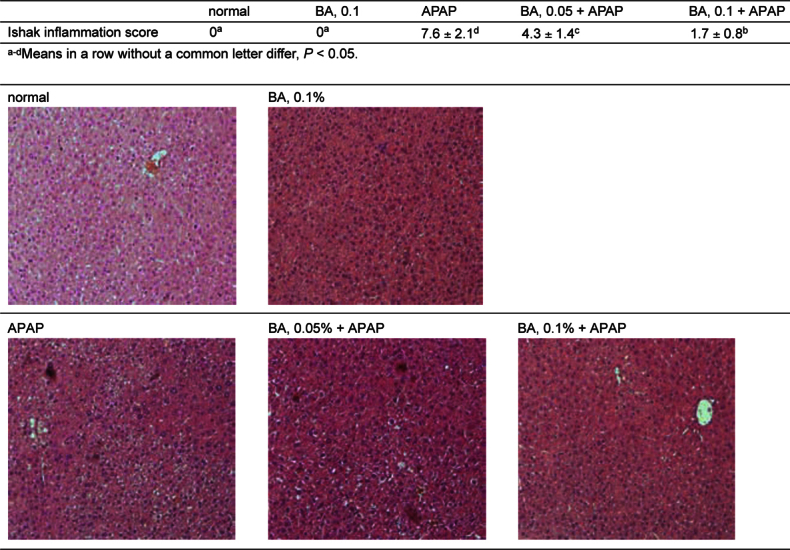
Effects of BA upon hepatic inflammation, determined by H&E stain, in mice with BA at 0, 0.05 or 0.1% for 4 weeks, and followed by APAP treatment. Normal groups had neither BA nor APAP treatments. BA groups had 0.1% BA intake without APAP treatment. Ishak inflammation score was used to quantify the injury area. Values are mean ± SD, n = 10. A representative image is shown for each group. Magnification: 200×.

Histological data revealed that BA intake at 0.1% only (without APAP) did not cause inflammatory stress and showed similar Ishak inflammation score as normal groups (Figure 6). APAP treatment led to multiple foci of inflammatory cell infiltration in liver, and increased Ishak inflammation scores (*P* & 0.05). BA intake at 0.05 and 0.1% decreased hepatic infiltration by inflammatory cells and lowered Ishak inflammation scores (*P* & 0.05), in which 0.1% BA treatment exhibited greater anti-inflammatory effects than 0.05% BA (*P* & 0.05).

**Table:** 

	normal	BA, 0.1	APAP	BA, 0.05 + APAP	BA, 0.1 + APAP
TNF-alpha	15 ± 2^a^	16 ± 3^a^	487 ± 23^d^	315 ± 18^c^	184 ± 10^b^
IL-6	19 ± 4^a^	17 ± 5^a^	412 ± 17^d^	294 ± 20^c^	147 ± 15^b^
MCP-1	16 ± 5^a^	12 ± 4^a^	503 ± 25^d^	324 ± 21^c^	160 ± 18^b^

^a-d Means in a row without a common letter differ, *P* < 0.05.^

## 4. Discussion

Our present study revealed that the pre-intake of BA, an active compound of *Boswellia* species, increased its deposit in liver and protected liver against subsequent APAP induced oxidative and inflammatory injury. Besides increasing GSH retention, we found that BA effectively decreased CYP2E1 activity and expression, lowered ROS, TNF-alpha and MCP-1 production, as well as suppressed HO-1, TLR-3, TLR-4, NF-κB p65 and p-JNK expression. In addition, our histological results indicated that BA improved diffuse ballooning degeneration and lobular inflammation caused by APAP. These findings support that BA is a potent hepatic protective agent.

APAP at high dose led to GSH depletion and GSSG accumulation [16]. Our data revealed that APAP decreased GR activity and expression, which definitely impaired the conversion from GSSG to GSH in liver. However, BA pre-intake maintained GR activity and expression, which contributed to convert GSSG to GSH. These results explained the greater GSH content and less GSSG level in livers of BA treated mice. HO-1 is an Nrf2 target and antioxidant responsive element-dependent gene, and the products of HO-1 catalyzed reactions could exert protective effects in liver against oxidative and noxious stimuli [17]. It is reported that the increased HO-1 expression promoted GSH synthesis and alleviated APAP induced hepatotoxicity [18]. Since BA supplement substantially retained hepatic HO-1 expression, it is reasonable to observe greater hepatic GSH homeostasis in BA treated mice. Obviously, BA benefited hepatic glutathione redox cycle through regulating GR and HO-1, which in turn enhanced antioxidative defense in liver. APAP stimulates hepatic CYP2E1, the major isozyme responsible for the formation of NAPQI, to cause ROS overproduction [19, 20]. It is reported that inhibition upon CYP2E1-mediated bio-activation could attenuate APAP-induced liver injury [21]. Our data indicated that BA pre-intake markedly suppressed subsequent APAP elicited CYP2E1 activity and protein expression, which consequently lowered ROS formation and diminished oxidative stress in liver. These findings revealed that BA, besides retaining hepatic GSH level, could provide other anti-oxidative actions like inhibiting CYP2E1 and maintaining HO-1 to protect liver against APAP.

APAP overdose activated NF-kB and JNK pathways through stimulating ROS generation [22, 23]. The activation of these signal pathways enhanced downstream inflammatory and oxidative reactions in liver and facilitated the production of cytokines and chemotactic factors like IL-6, TNF-alpha and MCP-1 [24, 25]. We found that BA pre-intake already lowered hepatic ROS level *via* its anti-oxidative activities, which definitely led to less stimulation for hepatic NF-kB activation and JNK phosphorylation. Our western blot data regarding hepatic protein expression of NFkB p65 and p-JNK agreed that BA intake at both doses limited the activation of these two signal pathways. Consequently, it is reasonable to observe lower hepatic levels of IL-6, TNF-alpha and MCP-1 in BA treated mice. The decrease in serum levels of ALT, AST and CRP in BA treated mice also agreed that BA intake declined APAP caused liver inflammatory stress. In addition, our histological data further supported that BA pre-intake effectively improved hepatic inflammatory injury.

TLRs play crucial roles in the pathological progression of inflammatory liver diseases because TLRs recognize endogenous damage-associated molecular patterns during inflammatory reactions [26]. TLR-3 is a cytoplasmic TLR, and TLR-4 is an extracellular TLR. The binding of endoligands like host cellular mRNA released from damaged cells to TLR-3 and/or TLR-4 promotes the expression of adaptor proteins including NF-κB, JNK and MyD88, which is followed by increasing cytokines and chemokines production [27, 28]. Zheng *et al*. [29] reported that APAP enhanced liver expression of TLR-3, TLR-4 and MyD88, and facilitated hepatic inflammatory reactions. Our western blot data agreed that TLR-3, TLR-4, NF-κB, JNK and MyD88 were involved in APAP caused hepatotoxicity. Furthermore, we found that BA pre-intake at 0.1% down-regulated hepatic expression of TLR-3 and TLR-4, which subsequently lowered the expression of adaptor proteins like MyD88, NF-κB and JNK; and finally weakened NF-κB and JNK mediated inflammatory response. Thus, the less formation of inflammatory factors such as TNF-alpha and MCP-1 in livers from BA treated mice could be explained. Apparently, BA could improve APAP-induced inflammatory injury by blocking upstream inflammatory mediators such as TLR-3 and TLR-4. However, it should be pointed out that BA intake at 0.05% failed to change protein expression of TLR-3, TLR-4 and MyD88, but effectively limited NF-κB p65 expression and JNK phosphorylation. Obviously, BA could directly decline NF-κB and JNK pathways to alleviate APAP-induced liver injury. These findings indicated that BA could provide multiple anti-inflammatory actions to ameliorate APAP-induced hepatic damage. Gayathri *et al*. [12] reported that BA exhibited anti-inflammatory activity in human PBMCs through inhibiting TNFalpha, IL-1beta and mitogen-activated protein kinase. Our present study provided *in vivo* anti-inflammatory data of BA, and extended BA’s mediating activity to TLRs and MyD88.

BA is naturally present in many edible plants including vegetables and herbs [13, 30]. The daily intake of BA at 0.1% in mice is approximately equal to 5.6 g for a 70-kg adult. Our data indicated that BA intake at this dose did not cause any toxic sign in mice. Thus, the application of this compound for prevention and/or alleviation of liver diseases might be safe and feasible. In addition, it is interesting to find that 0.05% BA intake did not lead to the deposit of BA intact form in liver, but still exhibited protective effects for liver against APAP. It is highly possible that the metabolites of BA also provide hepatic protective activities.

## 5. Conclusion

Boswellic acid pre-intake protected mice liver against subsequent acetaminophen-induced oxidative and inflammatory injury *via* maintaining hepatic GSH content, retaining activity and expression of GR and HO-1, decreasing inflammatory cytokines and suppressing protein expression of CYP2E1, NF-κB p65, JNK, TLR-3 and TLR-4. These findings support that boswellic acid was a potent hepatic protective agent.
