# Correction notice to: Alleviative effects from boswellic acid on acetaminophen-induced hepatic injury: Biomedicine (Taipei). 2016 Jun; 6 (2): 9. DOI: 10.7603/s40681-016-0009-1

**DOI:** 10.1051/bmdcn/2017070107

**Published:** 2017-03-07

**Authors:** Lung-Che Chen, Li-Hong Hu, Mei-Chin Yin

**Affiliations:** 1 Department of Otolaryngology, Taipei Medical University Hospital Taipei 110 Taiwan; 2 Shanghai Research Center for the Modernization of Traditional Chinese Medicine, Shanghai Institute of Materia Medica, Chinese Academy of Sciences Shanghai 201203 China; 3 Department of Nutrition, China Medical University Taichung 404 Taiwan

Wrong histological pictures were shown in original Fig. 6 of this article because authors carelessly selected these pictures from a wrong picture file, which belongs to another study. Authors apology for this error.

The following histological photos are correct for Fig. 6 of this article. Please use these correct photos to replace those old ones shown in original Fig. 6 of this article.

**Fig. 6:**
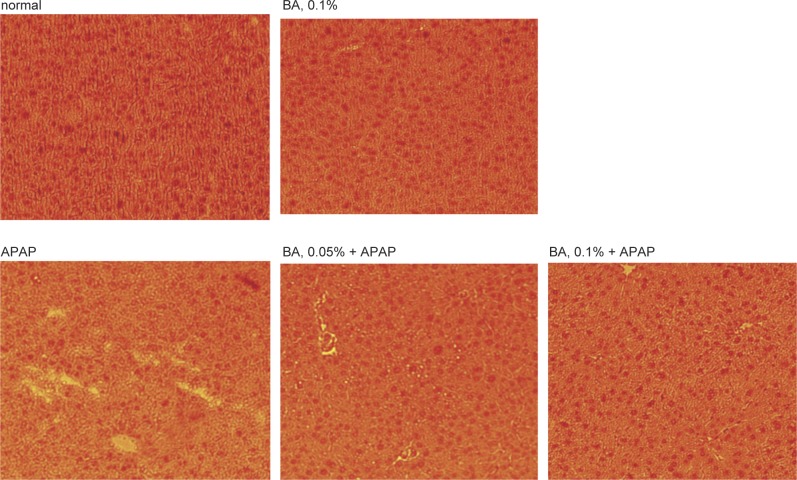
Effects of BA upon hepatic inflammation, determined by H&E stain, in mice with BA at 0, 0.05 or 0.1% for 4 weeks, and followed by APAP treatment. Normal groups had neither BA nor APAP treatments. BA groups had 0.1% BA intake without APAP treatment. A representative image is shown for each group. Magnification: 200×

